# Simplifying External Load Data in NCAA Division-I Men's Basketball Competitions: A Principal Component Analysis

**DOI:** 10.3389/fspor.2022.795897

**Published:** 2022-02-16

**Authors:** Jason D. Stone, Justin J. Merrigan, Jad Ramadan, Robert Shaun Brown, Gerald T. Cheng, W. Guy Hornsby, Holden Smith, Scott M. Galster, Joshua A. Hagen

**Affiliations:** ^1^Human Performance Innovation Center, School of Medicine, Rockefeller Neuroscience Institute, West Virginia University, Morgantown, WV, United States; ^2^College of Physical Activity and Sport Sciences, West Virginia University, Morgantown, WV, United States; ^3^Men's Basketball, Athletics Department, West Virginia University, Morgantown, WV, United States

**Keywords:** sport science, wearables, inertial measurement unit (IMU), collegiate athletics, athlete monitoring

## Abstract

The primary purpose was to simplify external load data obtained during Division-I (DI) basketball competitions via principal component analysis (PCA). A secondary purpose was to determine if the PCA results were sensitive to load demands of different positional groups (POS). Data comprised 229 observations obtained from 10 men's basketball athletes participating in NCAA DI competitions. Each athlete donned an inertial measurement unit that was affixed to the same location on their shorts prior to competition. The PCA revealed two factors that possessed eigenvalues >1.0 and explained 81.42% of the total variance. The first factor comprised total decelerations (totDEC, 0.94), average speed (avgSPD, 0.90), total accelerations (totACC, 0.85), total mechanical load (totMECH, 0.84), and total jump load (totJUMP, 0.78). Maximum speed (maxSPD, 0.94) was the lone contributor to the second factor. Based on the PCA, external load variables were included in a multinomial logistic regression that predicted POS (Overall model, *p* < 0.0001; AUC_centers_ = 0.93, AUC_guards_ = 0.88, AUC_forwards_ = 0.80), but only maxSPD, totDEC, totJUMP, and totMECH were significant contributors to the model's success (*p* < 0.0001 for each). Even with the high significance, the model still had some issues differentiating between guards and forwards, as in-game demands often overlap between the two positions. Nevertheless, the PCA was effective at simplifying a large external load dataset collected on NCAA DI men's basketball athletes. These data revealed that maxSPD, totDEC, totJUMP, and totMECH were the most sensitive to positional differences during competitions. To best characterize competition demands, such variables may be used to individualize training and recovery regimens most effectively.

## Introduction

Sport science applications in collegiate basketball synergize sport coach knowledge and data-driven scientific strategies to individualize player training and recovery regimens. The goal is to facilitate beneficial physiological adaptations to enhance in-game performances. The data most commonly collected for the aforementioned purposes include, but are not limited to, external player loads via player trackers [e.g., radio frequency identification (RFID), inertial measurement units (IMU)], internal player loads *via* wearables [e.g., heart rate monitors via electrocardiography (ECG) straps], internal player loads via subjective ratings of perceived exertion, subjective wellness, sleep monitoring via wearables or self-report, neuromuscular performance (e.g., force plate testing), as well as basketball-specific performance (e.g., shooting percentages, efficiency ratings) and tactical schemes (e.g., drill and play-call selections) (Fox et al., 2017; Edwards et al., 2018; Svilar and Jukić, 2018). From these data, daily individual player preparedness reports are generated and disseminated to the coaching staff via the performance and sports medicine personnel (i.e., sport scientist, strength and conditioning coach, nutritionist, athletic trainer, physical therapist, team physician). More specifically, these analyses often comprise comparisons within each athlete's objective and subjective external and internal training and recovery statuses, as well as group comparisons between positions (POS; e.g., guards, forwards, centers), player statuses (e.g., starters, reserves), and/or competition outcomes (e.g., Wins and Losses) (Parmar et al., 2018; Svilar et al., 2018; Svilar and Jukić, 2018; Bunker and Thabtah, 2019; Rojas-Valverde et al., 2019; Russell et al., 2020). When these data are valid and reliable, they may be automated and actioned to closely monitor physiological and psychological demands during training and competitions with a high degree of individualization. By identifying strengths and weaknesses of individual athletes and the team, the quantification of training and competition demands provide enhanced contextual feedback on athlete performance and recovery that subsequently enables improved individualized programming efforts. Pertaining to applied performance and sport scientists, data-driven performance monitoring is helpful for purposeful organizing (and individualizing) within their athlete monitoring framework.

However, despite rigorous pursuit by practitioners and researchers, the identification of key metrics for athlete monitoring purposes continues to be a difficult problem. The expansion of wearables in the sport industry inherently inflates the number of monitoring variables, with data now being systematically sampled at high rates at the individual athlete level from technologies such as RFID, IMU, ECG, global positioning systems (GPS), local positioning systems, accelerometers, or some combination of multiple sensors (Taylor et al., 2012). Consequently, these automated collections create large individual athlete data sets, often making it even more challenging to identify the most appropriate monitoring variables within a given team. Additionally, as new technologies are developed and commercialized, end users are left with limited understanding regarding the metrics reported until further scientific investigations are executed.

One statistical approach that assists in simplifying datasets in team-sports and other high-performance environments (e.g., tactical) is a principal component analysis (PCA), which is an effective strategy for making athlete monitoring datasets more actionable for practitioners (O'Donoghue, 2008; Federolf et al., 2014; Laffaye et al., 2014; Stein et al., 2017; Parmar et al., 2018; Svilar et al., 2018; Rojas-Valverde et al., 2019, 2020; Merrigan et al., 2021; Terner and Franks, 2021). In general, a PCA reduces the number of dimensions in a relatively large dataset by identifying variables that possess low degrees of multicollinearity and are the most responsible for fluctuations in overall variance (typically at least 70–80% of the total variance explained) (Rojas-Valverde et al., 2020). Previous research in professional 1st Spanish Division basketball athletes deployed a series of PCA's for Guards, Forwards, and Centers and determined that the training load demands differed across POS (Svilar et al., 2018). For example, high intensity and total counts of accelerations explained reasonable amounts of variance in Centers but not Guards and Forwards, whereas high intensity and total counts of decelerations appeared to be more relevant. Although that study demonstrated clear differences in external load demands during training itself for basketball position groups, the analysis did not contain competition data, which is imperative for the sake of training specificity. Still, those findings suggest that player monitoring tactics (i.e., selection of metrics for monitoring and reporting) likely vary based on the different POS at the elite level of basketball. Similarly, PCAs were utilized in college and professional rugby, American football, basketball, baseball, volleyball, and soccer (Parmar et al., 2018; Casamichana, 2019), with recent proposals and systematic reviews asserting a need for more PCAs in sport settings (Stein et al., 2017; Rojas-Valverde et al., 2019, 2020). Provided the situational specificity of individualized athlete monitoring, continual research incorporating PCAs into other team-sport applications, such as collegiate athletics, is certainly warranted. To practically apply PCA results in these types of settings, one might consider how well (or not) the variables contained within the reduced dimensions predict various sport-specific contexts of interest, such as position group assignment, home and away competitions, opponent difficulty (e.g., Power 5 conference opponent or not), regular and postseason competitions, and/or player statuses (e.g., starter or reserve) (Fowler et al., 2017; Staunton et al., 2018; Fox et al., 2019, 2021). Depending on the intended outcome from analysis, in these instances a multinomial logistic regression may be preferred in lieu of alternative methods (e.g., discriminant analysis) because the focal point may be the predictor variables themselves and the prevention of overfitting such that analytical insights were more applicable to outside applications (e.g., other teams, researchers) (Luo et al., 2011). Moreover, a sport scientist might be interested in determining which external and/or internal load metrics are the most sensitive to group classification (i.e., how did the predictor variables change between POS groups) rather than solely being interested in the prediction outcome itself (i.e., which POS group did the model assign an athlete to). Indeed, the latter likely justifies the utilization of discriminant analysis, such as an instance in which a coach is trying to decide which POS group a new athlete might be most suitable for during training. However, when the primary question pertains to how the load demands vary between POS group (or home/away, Power 5/not, starter/non-starter), logistic regression may be more suitable.

Therefore, the primary purpose of this study was to utilize PCA to simplify external loadmetrics from IMU data that were collected during competitions across a single season of NCAA Division-I (DI) men's basketball in a Power-5 conference team. The secondary purpose was to examine whether there were discernible player POS differences in the most pertinent metrics identified in the PCA.

## Methods

All athletes signed the institutionally approved informed consent document. All procedures were approved by West Virginia University's Institutional Review Board (#2102249143).

### Subjects

Ten NCAA DI men's basketball athletes (Mean ± SD; *n* = 10; height: 196.09 ± 8.01 cm; weight: 96.95 ± 11.14 kg) were included in the present study. The athletes were partitioned into three POS groups as these were dictated by the sport coaching staff at the start of the season based on team playing style (Guards: *n* = 4; height = 188.60 ± 4.34 cm; weight = 85.98 ± 3.24 kg; Forwards: *n* = 3; height = 192.27 ± 5.87 cm; weight = 94.72 ± 4.78 kg; Centers: *n* = 4; height = 204.89 ± 3.88 cm; weight = 113.80 ± 3.33 kg). These positions also align with previous research in basketball athletes (Svilar et al., 2018).

### Experimental Design

To examine the external load demands that are characteristic for NCAA DI men's basketball competitions, a retrospective study design was utilized following a single competitive season. Prior to competitions, the IMUs were positioned in a holster that was stitched into the uniform shorts and located near the posterior superior iliac spine on the right side for each athlete. These holsters were constructed in collaboration between the sensor manufacturers and team equipment managers to ensure unnecessary movement of the sensors was negligible and positioning was consistent throughout the season. Data were obtained from a wearable IMU sensor and then compiled into a central database after each competition. Following the season conclusion, data were de-identified, exported, and analyzed for the purposes of identifying KPIs specific to basketball competitions.

### Protocol

External load data from wearable IMU sensors (KINEXON Precision Technologies, version 1.0, Munich, Germany) were collected, at 20 Hz, from each athlete during 27 competitions interspersed throughout the 2020-2021 NCAA Men's Basketball season. A previous study examining the validity of this system reported an average total typical error of estimates to be 2.5% (± 1.5%) when five adult male team sport amateur athletes performed a variety of movements comprising walking, jogging, and sprinting of different distances, as well as changes of direction and jumping (Alt et al., 2020). All system installations and calibrations were performed by the same technician prior to the season starting. Competitions comprised 24 regular season games, one game from the conference tournament, and two games from the NCAA tournament. A total of 221 observations of players in each game were recorded, which were further broken down into 64, 75, and 82 cases for Centers, Forwards, and Guards, respectively.

Session recordings occurred throughout each game-day and were initiated and ceased at the same time for each athlete. Individual phase recordings were time stamped and segmented into Shoot around, Warm-Up, 1st Half, Half-Time, and 2nd Half phases. However, for the sake of this study, the dataset that was analyzed only included external load data obtained during the active competition minutes (i.e., during the 1st Half and 2nd Half). The 1st Half recording was initiated immediately prior to the referee throwing up the ball to signify the “tip-off” and concluded upon the sound of the buzzer when the game clock reached zero. A separate half-time phase was generated to account for the time spent in the locker room to partition the data from the 1st and 2nd Halves. The 2nd Half session began as soon as a team took possession and the game clock started counting down. Similar to the 1st Half, this phase concluded when the buzzer sounded, and the game clock reached zero. Any overtime periods were excluded from analysis as the scope of this study was to merely examine typical competitions. The variables of interest encapsulated summated mechanical loads (totMECH; a.u.), jump loads (totJUMP; J), accelerations (totACC; count), and decelerations (totDEC; count) from the 1st and 2nd Half, as well as the average speed (avgSPD; mph) and maximum speed (maxSPD; mph) from the entire game. Mechanical loads were calculated utilizing a proprietary equation that placed acceleration and deceleration events into multiple, weighted intensity bins that were collectively summated into a single value (i.e., totMECH) for a given phase (e.g., halves). The totACC and totDEC were identified using a threshold of 1.5 m/s^2^ and a minimum duration 0.5 s as dictated by the proprietary software. The total jump loads (totJUMP) were calculated by multiplying the player's body mass by the gravity constant (9.8 m/s), and the jump height in meters for each jump event. Then, all jump loads were summated to provide the totJUMP, as a volume-load metric describing the cumulative intensity and volume of jumps for the session. For a movement to be considered a jump, an athlete had to elevate for a minimum airtime of 0.3 s.

### Statistical Analysis

Data were collected, stored, and exported from Kinexon before being imported and prepared for analysis in R Version 4.0.3 (Team, 2019). The Kinexon companion software exports a total of 109 external load variables, which are left for practitioners to determine which are the most useful. These variables comprise event counts (e.g., number of accelerations, decelerations, jumps, sprints), intensity bandings (e.g., acceleration zones 1–4), and durations (e.g., time spent in speed zones) of both two-dimensional (2D) and three-dimensional (3D) movements. Since many of these variables are redundant and utilized as contextual information (e.g., durations and distances covered over certain speed zones, counted numbers of accelerations/decelerations in different intensity zones) to help characterize primary summary variables (e.g., mechanical load, jump load, total counts), most of them were omitted from analysis to prevent high degrees of multicollinearity and improve overall sampling adequacy.

To ensure data adequacy for factor analysis, a measure of sampling adequacy (MSA) was derived for the entire data set *via* the Kaiser-Meyer-Olkin measure (KMO). The overall MSA was meritorious at 0.81; thus, providing sufficient confidence to conduct the PCA (Kaiser, 1960; Kaiser and Little, 1974). Bartlett's Test of Sphericity was also conducted and confirmed that the correlation and identity matrices were divergent (i.e., the variables included were correlated enough to provide practical value without redundancy in the data), further supplying confidence that dimension reduction was suitable (*p* < 0.0001). A PCA on correlations was calculated to identify the principal components accounting for the most relevant variance, which was dictated by those possessing eigenvalues > 1.0. From there, a variable loading matrix with a *VariMax* rotation was generated to identify variables in the principal components with an individual loading value ≥ 0.70. Next, a multinominal logistic regression was constructed using athlete POS (Guards, Forwards, Centers) as the response variable, and the most relevant PCA variables as predictors. This helped to better understand POS differences (i.e., which position was higher or lower with respect to each predictor) with the variables identified in the PCA as having a loading ≥ 0.70. With this regression, an odds ratio (OR) was calculated for each predictor with respect to a Guards to Forwards comparison, as well as a Guards to Centers comparison. The areas under the receiver operating characteristics (ROC) curves were also reported. These ROC curves were meant to depict the probability that the external load variables could correctly assign athletes to the correct POS group. Lastly, one-way analysis of variance (ANOVA) tests were conducted for those variables that significantly increased the odds of accurately assigning an athlete to their correct POS group in the regression. Following significant univariate effects, Tukey's HSD *post-hoc* analysis was carried out to ascertain differences between individual POS groups. Cohen's *d* was used to calculate effect size with thresholds as follows: trivial: 0.0–0.19; small: 0.20–0.49; moderate: 0.50–0.79; large: ≥ 0.80 (Cohen, 1988). All alpha levels were set at *p* < 0.05 and statistical analysis was conducted in JMP Pro Version 16 (Jones and Sall, 2011).

## Results

### Simplifying External Load Data

The first (eigenvalue = 3.80; % variance = 63.45%) and second (PC2; eigenvalue = 1.07; % variance = 17.85%) factors explained 81.30% of the total variance in athletes' external loads during basketball competitions. The third factor only possessed an eigenvalue of 0.43 and thus was omitted from further analysis, according to Kaiser criterion.

**Figure 1:**
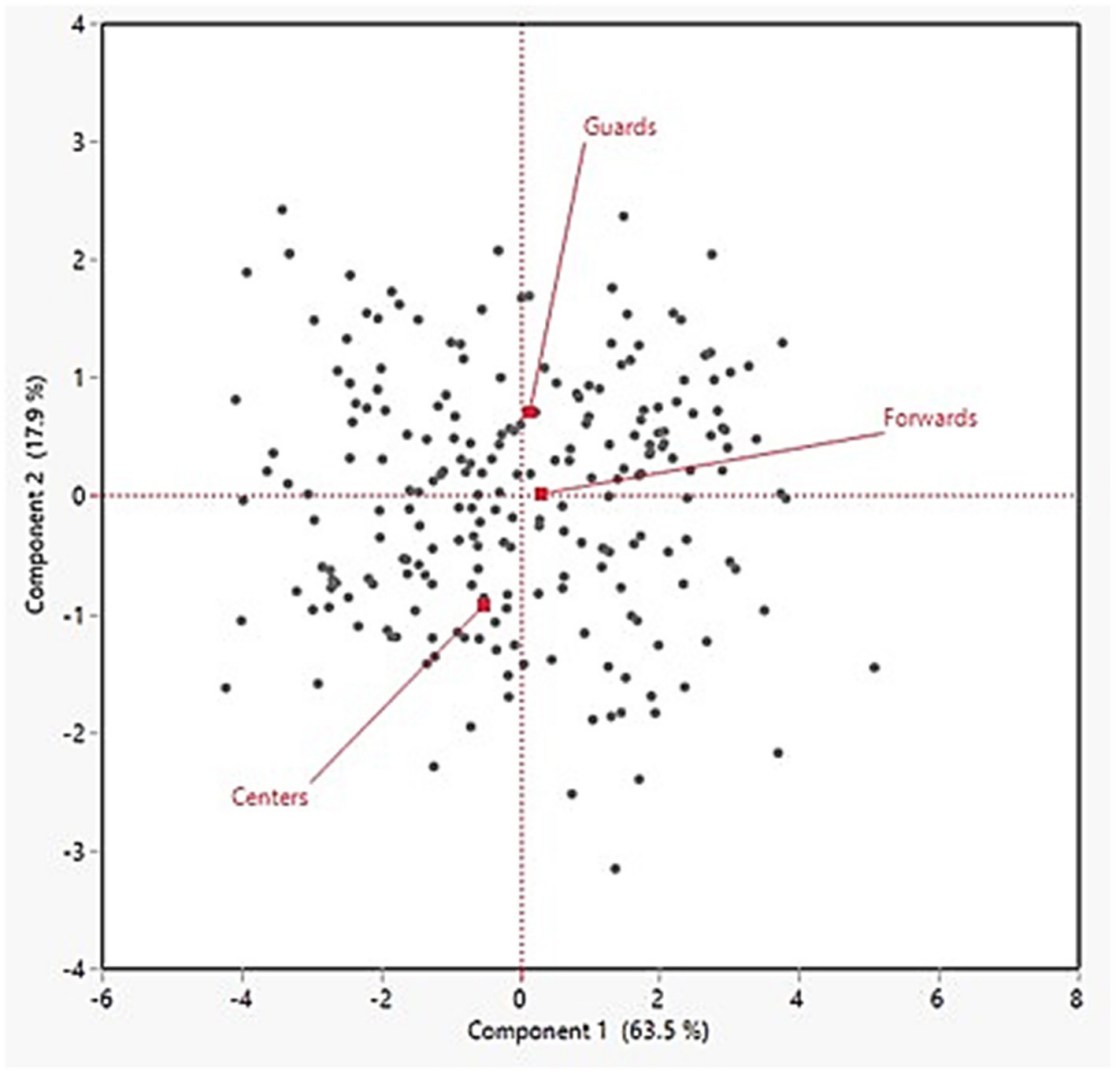
Individual observations derived from the principal component analysis and supplemented with the player position variable.

**Figure 2:**
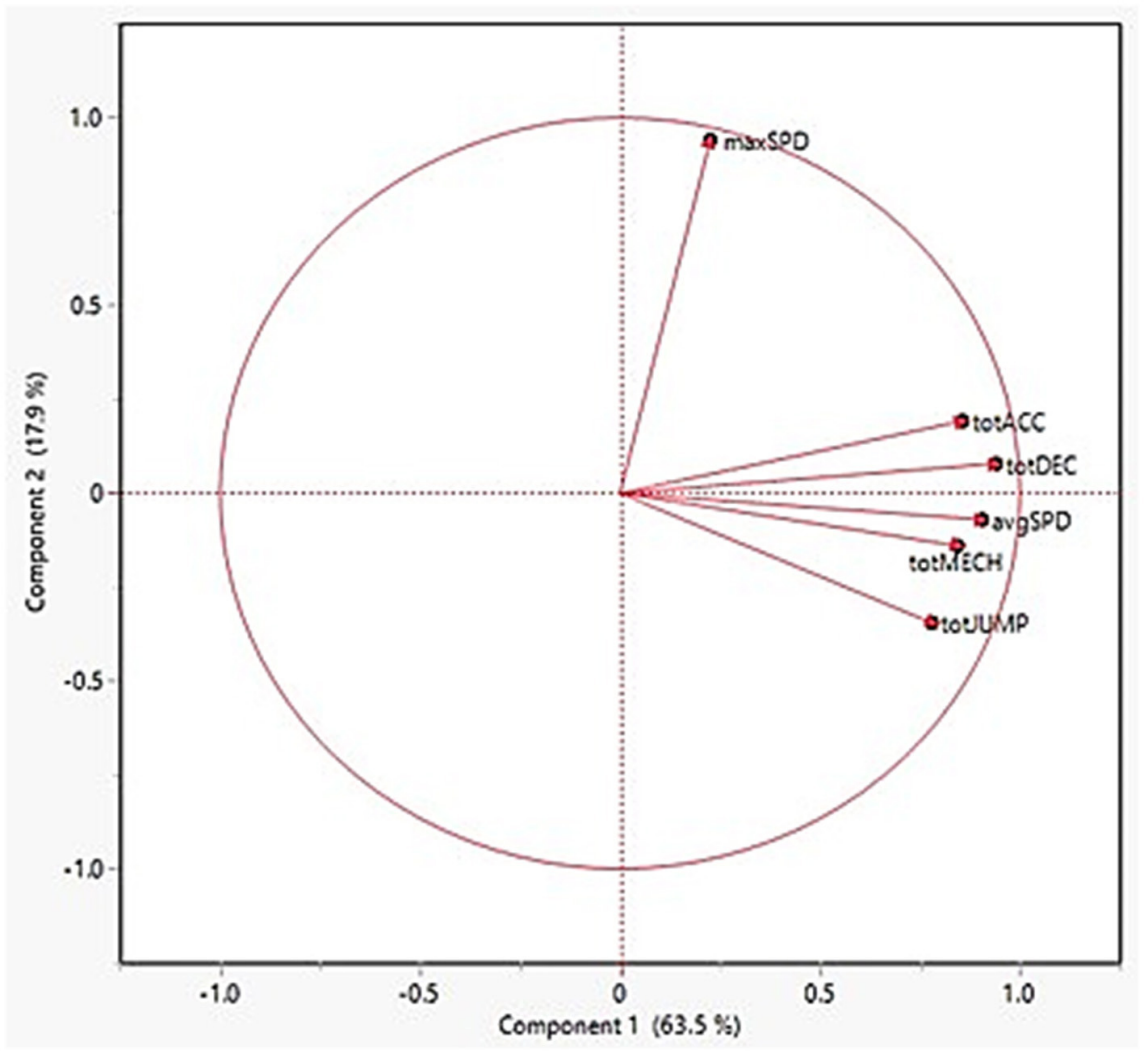
Loading plot for principal components 1 and 2. Total mechanical load, totMECH; jump load, totJUMP; accelerations, totACC; decelerations, totDEC; average speed, avgSPD; maximum speed, maxSPD.

A loading matrix for the first two factors was created to only report instances where the loading magnitude was ≥ 0.70. Additionally, summary plots for the individual observations (supplemented by POS) and rotated loading factors are found in Figures 1, 2, respectively. In Figure 1, the three POS groups (Guards, Forwards, Centers) appear to distance themselves from each other, implying that there are distinct differences in the external load demands among them. In the first factor, the main contributors included five of the six external load variables with the following relative loadings: totDEC, 0.94; avgSPD, 0.91; totACC, 0.86; totMECH, 0.84; totJUMP, 0.78. However, maxSPD was the sole main contributor, with a relative loading of 0.94, in the second factor thereby suggesting maxSPD possesses a low correlation with the PC1 variables. The loading plot further illustrates this as the five variables in PC1 are relatively “clustered” together whereas the loading arrow for maxSPD clearly distinguishes itself from the rest.

### Predicting Player Positions Based on External Load

**Figure 3:**
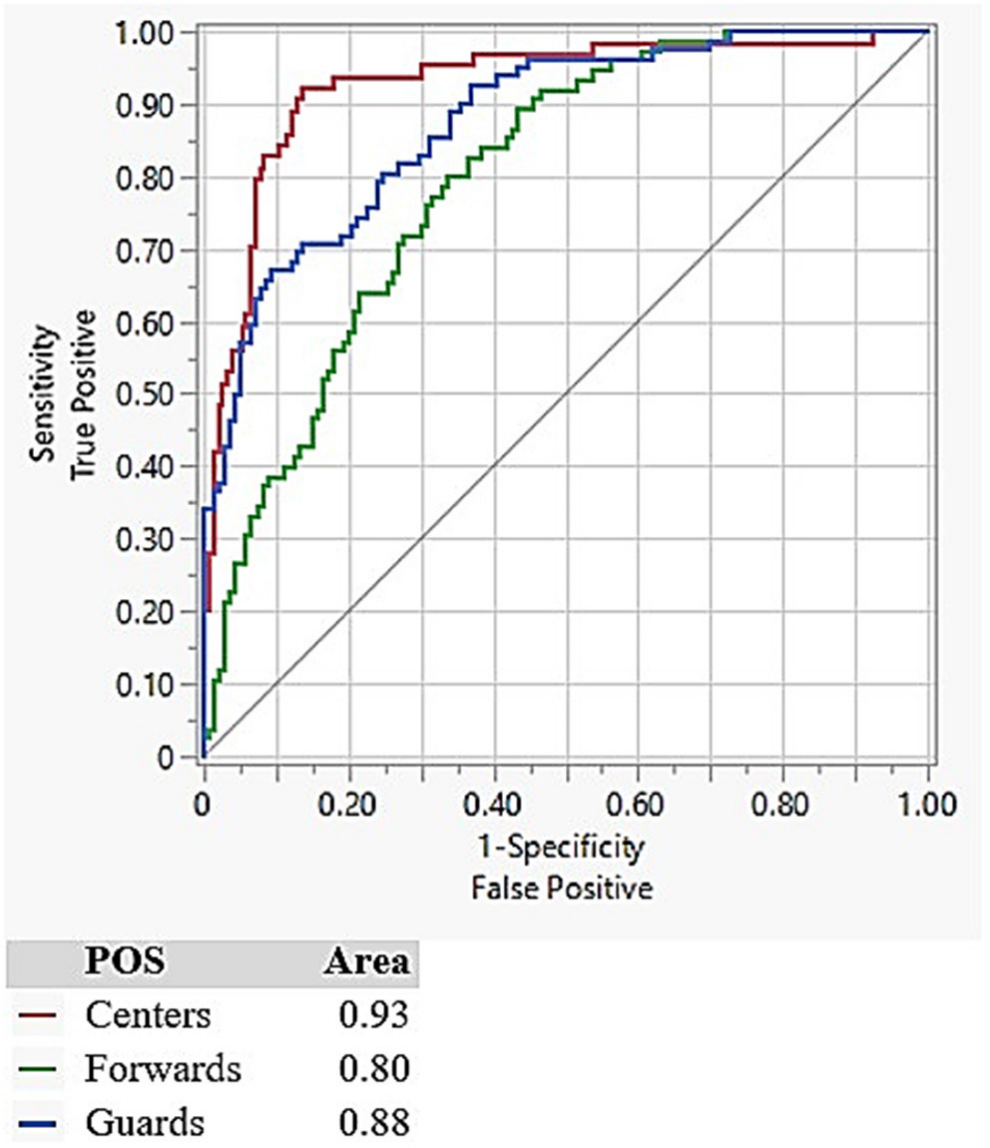
Receiver operating characteristic curves for each of the athlete position groups.

The overall model was significant at assigning athletes to POS (*p* < 0.0001; AICc = 322.15; BIC = 367.69), with maxSPD, totJUMP, totDEC, and totMECH each contributing to the model's successful POS assignment (*p* < 0.0001; Table 1). The confusion matrix (Table 2) details that Centers, Guards, and Forwards were correctly assigned in 82.8, 70.7, and 54.7% of model iterations, respectively. The greatest predictive error appeared when Forwards were labeled as Guards, which occurred nearly one-third of the time. The differences in prediction accuracy for each POS was further confirmed by ROC curves, which revealed the greatest area under the curve for Centers (0.93), followed by Guards (0.88) then Forwards (0.80) (Figure 3).

**Table 1:** Summary parameter estimates from a multinomial logistic regression on external load variables.

**Term**	**Estimate ±SE**	**Exp(β)**	**Exp(β) CI_**95%**_**	**ChiSquare**	***p*-value**
**Guards to Centers**					
Intercept	29.28 ± 4.43			43.71	<0.0001^*^
totJUMP	0.00033 ± 0.000071	1.00033	(1.000191, 1.000469)	21.67	<0.0001^*^
totMECH	−0.00069 ± 0.00062	0.99931	(0.998097, 1.000525)	1.23	0.27
totDEC	−0.013 ± 0.0067	0.98708	(0.974206, 1.000132)	3.99	<0.05^*^
totACC	0.0028 ± 0.0045	1.00280	(0.993998, 1.011688)	0.37	0.54
avgSPD	−1.24 ± 2.44	0.28938	(0.002424, 34.549739)	0.26	0.61
maxSPD	−4.23 ± 0.69	0.01455	(0.003764, 0.056270)	37.34	<0.0001^*^
**Guards to Forwards**					
Intercept	16.56 ± 3.43			23.34	<0.0001^*^
totJUMP	0.0000016 ± 0.000051	1.00000	(0.999902, 1.000102)	0.00	0.98
totMECH	−0.0026 ± 0.00065	0.99740	(0.996133, 0.998675)	15.56	<0.0001^*^
totDEC	0.020 ± 0.0049	1.02020	(1.010450, 1.030047)	16.48	<0.0001^*^
totACC	−0.0044 ± 0.0033	0.99561	(0.989191, 1.002070)	1.74	0.19
avgSPD	0.72 ± 2.04	2.05443	(0.037689, 111.988927)	0.12	0.73
maxSPD	−2.64 ± 0.52	0.07136	(0.025753, 0.197740)	25.44	<0.0001^*^

*SE, standard error; Exp(β), exponentiation of the β coefficient; Exp(β)CI_95%_, 95% confidence intervals; total mechanical load, totMECH; jump load, totJUMP; accelerations, totACC; decelerations, totDEC; average speed, avgSPD; maximum speed, maxSPD.*

*The estimates are the log odds of moving from baseline (Guards) to either Centers or Forwards. Ex. For every increase in totJUMP the odds of being predicted as a Center increase since the estimate is positive. However, every unit increase in totDEC and maxSPD lead to a lower likelihood of being predicted as Centers from Guards.*

* *Denotes statistical significance at p < 0.05*.

**Table 2:** A confusion matrix from a multinomial logistic regression on external load variables.

	**Actual N**	**Predicted count**		
**Position**		**Centers**	**Forwards**	**Guards**
Centers	64	53	9	2
Forwards	75	13	41	21
Guards	82	3	21	58

### Differences in External Load KPI's for Player Positions

**Figure 4:**
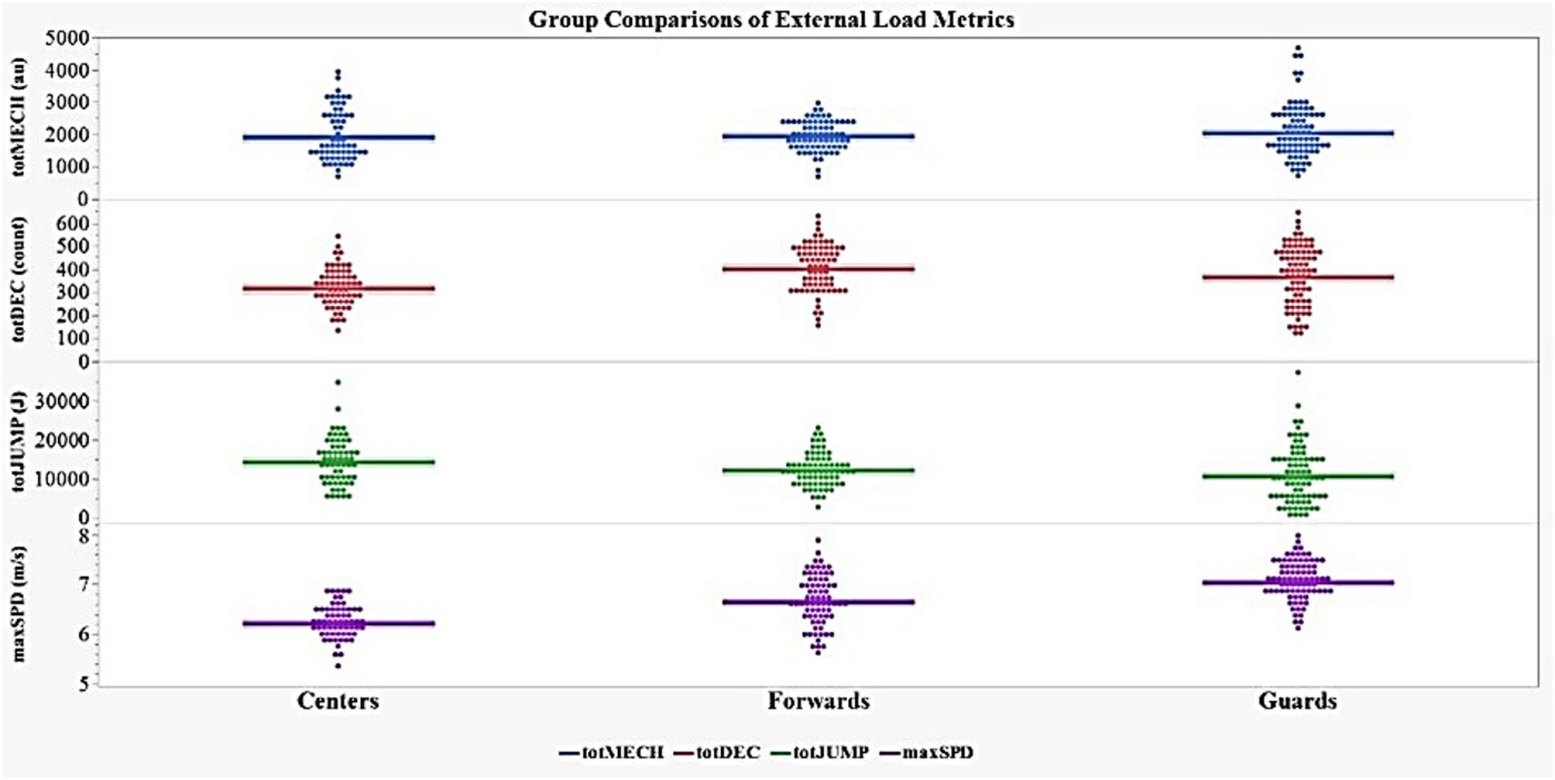
Group comparisons of external load variables by player position. maxSPD, maximum speed; totDEC, total decelerations; totJUMP, total jump load.

Descriptive statistics of each external load variable, separated by POS, are displayed in Table 3. There were significant differences among POS for maxSPD [*F*_(2, 218)_ = 66.06, *p* < 0.0001], totDEC [*F*_(2, 218)_ = 11.52, *p* < 0.0001], and totJUMP [*F*_(2, 218)_ = 5.28, *p* < 0.01], but not totMECH (Figure 4).

**Table 3:** Primary external load variables for Centers (*n* = 64), Forwards (*n* = 75), Guards (*n* = 85).

**Variable**	**POS**	**Mean ±SD**	**SE Mean**	**CI_**95%**_**
maxSPD (m/s)	Centers	6.23 ± 0.33	0.04	(6.14, 6.31)
	Forwards	6.63 ± 0.52	0.06	(6.51, 6.75)
	Guards	7.04 ± 0.39	1.04	(6.95, 7.12)
totJUMP (J)	Centers	14009.34 ± 6017.65	752.21	(12506.17, 15512.50)
	Forwards	11960.21 ± 4324.49	499.35	(10965.24, 12955.19)
	Guards	10753.27 ± 7249.65	800.59	(9160.34, 12346.19)
totDEC (count)	Centers	307.95 ± 87.27	10.91	(286.15, 329.75)
	Forwards	397.04 ± 106.45	12.29	(372.55, 421.53)
	Guards	362.32 ± 126.38	13.96	(334.55, 390.09)
totMECH (au)	Centers	1853.31 ± 814.06	101.76	(1649.97, 2056.66)
	Forwards	1907.11 ± 480.79	55.52	(1796.49, 2017.73)
	Guards	2034.63 ± 848.26	93.67	(1848.29, 2221.06)

*POS, position; SD, standard deviation; SE Mean, standard error mean; CI_95%_, 95% confidence intervals; totJUMP, total jump load; totDEC, decelerations; maxSPD, maximum speed*.

Guards possessed significantly higher maximum speeds than Forwards and Centers (*p* < 0.0001) and Forwards were significantly faster than Centers (*p* < 0.0001). All three of these comparisons also reported large effect sizes (≥ 0.90). Forwards (*p* < 0.0001) and Guards (*p* < 0.01) performed significantly more totDEC compared to Centers, although Forwards and Guards did not differ in the totDEC performed. The observed effect size comparing Forwards to Centers was large with a near medium effect size for Guards and Centers and a small effect size for Forwards and Guards. With respect to totJUMP, Centers produced significantly greater total jump loads (measured in arbitrary units) than Guards (*p* < 0.01; near medium effect size) but there were no differences between Centers and Forwards or Forwards and Guards (small effect sizes). A summary table of the results from the *post-hoc* analysis is presented in Table 4.

**Table 4:** Tukey's HSD *post-hoc* ordered differences pairwise comparisons for the external load demands of different player positions.

**Variable**	**Group 1**	**Group 2**	**Difference ±SE**	**CI_**95%**_**	***p*-value**	**Cohen's d**
maxSPD	Guards	Centers	0.81 ± 0.07	(0.65, 0.98)^*^	<0.0001^*^	2.22
	Guards	Forwards	0.41 ± 0.07	(0.25, 0.57)^*^	<0.0001^*^	0.90
	Forwards	Centers	0.40 ± 0.07	(0.23, 0.57)^*^	<0.0001^*^	0.90
totDEC	Forwards	Centers	89.09 ± 18.63	(45.13, 133.04)^*^	<0.0001^*^	0.91
	Guards	Centers	54.36 ± 18.26	(11.28, 97.45)^*^	<0.01^*^	0.49
	Forwards	Guards	34.72 ± 17.49	(−6.55, 75.99)	0.12	0.30
totJUMP	Centers	Guards	3256.07 ± 1005.49	(883.21, 5628.93)^*^	<0.01^*^	0.48
	Centers	Forwards	2049.12 ± 1025.86	(-371.79, 4470.04)	0.12	0.40
	Forwards	Guards	1206.95 ± 963.19	(−1066.08, 3479.97)	0.42	0.20

* *Denotes statistical significance at p < 0.01; POS, position; SD, standard deviation; SE Mean, standard error mean; CI_95%_, 95% confidence intervals; totJUMP, total jump load; totDEC, decelerations; maxSPD, maximum speed*.

## Discussion

The specific aims of the present study were initially to utilize PCA to simplify external load in collegiate basketball athletes. From there, the objective was to utilize the PCA results to ascertain whether key external load metrics were sensitive to varying POS demands during competition. By reducing the number of dimensions in large datasets, as often experienced in high performance environments, staff can focus on a small collection of variables for individualized daily athlete monitoring. Undoubtedly, this is a preferred framework in comparison to sifting through hundreds of variables after each session, as the latter is far too cumbersome when trying to make routine, data-driven decisions and/or automate portions of the daily monitoring analysis (Rojas-Valverde et al., 2020). Subsequently, performance staff and sport coaches are better able to adjust training designs and game strategies for desirable performance enhancements that are based on quantitative insights. The present study utilized PCA to reduce the dimensions of external load data collected during collegiate basketball competitions, which simplified the dataset to two factors that helped differentiate player POS. In sport, coaches and practitioners often apply the approach of training specificity to their programming, with the primary goal being to sufficiently prepare athletes for the physical and psychological demands of competition (Schelling and Torres-Ronda, 2016). As such, for training to be as specific as possible, one must first consider the demands for which they are preparing for, which is obtained through analyses of different competition scenarios (i.e., competition levels, across seasons, sports, genders). Then, coaches and practitioners may work backwards as they begin to develop micro-, meso-, and macro-cycle periodization plans based upon competition demands to subsequently augment recovery and performance while mitigating injury and burnout risk (Schelling and Torres-Ronda, 2016; Cunanan et al., 2018; Stone et al., 2021). The PCA (and follow-up analyses) contained herein follows this train-of-thought as discernible differences between POS in basketball athletes were recognized using only a few of the most pertinent variables that were sensitive to variations in competition demands.

The totDEC possessed the largest loading in the first factor, which is similar to previous findings from PCA on international professional-level basketball athletes' external load data (Svilar et al., 2018). Although the aforementioned study conducted separate PCAs for each POS group and only focused on the load demands of in-season training, not competitions, total decelerations were revealed as one of the most important metrics for each POS. These similarities in findings likely suggest that a large amount of braking motor actions occur in response to the majority of movements performed during basketball. Examples include high-intensity bursts of acceleration and changes of direction, as well as rapidly altering pacing strategies, such as when a ball handler is attempting to deceive and surpass a defender (Schelling and Torres-Ronda, 2016). The high volume of decelerations may become difficult to manage as high volumes of these eccentric actions typically elicit skeletal muscle damage (Howatson and Milak, 2009). Resultantly, neuromuscular functioning may be impaired (if not properly managed), so close monitoring of daily fluctuations in such movements is crucial to ensuring desirable training adaptations are achieved rather than overtraining. Based on the repeated bout effect, athletes may better prepare for these volumes of decelerations during basketball competition by slowly progressing training strategies that optimize eccentric loading (i.e., greater eccentric velocities and power with lower exercise induced muscle damage) (Merrigan and Jones, 2021), as well as the elastic properties that maximize the utilization of the stretch-shortening (Gual et al., 2016; Schelling and Torres-Ronda, 2016).

Of course, a comprehensive training regimen for basketball athletes extends well-beyond the sole purpose of enhancing declarative ability. High performance in basketball necessitates high capacities of executive function and psychomotor performance, muscular strength and endurance, range of motion, and, more specifically, sprinting, changing of directions, pace (i.e., accelerating and decelerating), and jumping (Schelling and Torres-Ronda, 2016). According to our findings and others, these demands likely differ per player POS (due to the individual roles/responsibilities of each athlete and POS group), thereby suggesting that training programs should be tailored to each POS and individualized to each athlete (Svilar et al., 2018). For example, Guards performed over 50 more decelerations on average during competition and reached significantly faster maximal speeds in comparison to Centers, while Centers generated significantly higher jump loads than Guards (over 3,000 J; Table 4). Centers are mainly tasked with playing around the rim on offense and defense (i.e., close-range shooting, rebounding, rim protecting) whereas Guards play around the perimeter (i.e., mid- and long-range shooting, on-ball defending). Therefore, it is conceivable that Centers, who are inherently larger bodied individuals than Guards, are doing more straight line running from rim-to-rim with a large proportion of their actions ending with a high-intensity jump; while Guards are more likely to engage in much more accelerations and decelerations as they navigate a larger area of the court at much faster speeds. Meanwhile, forwards are not as easy to dissociate, as they typically perform hybrid roles between Guards and Centers. Often, these athletes are similar in stature to Centers but similar in playing style to Guards. Indeed, the present multinomial logistic regression predicted player POS using the PCA variables with remarkable success, except for assigning Forwards to the correct group. More specifically, Forwards were wrongfully labeled as Guards more than 25% of the time and as Centers nearly 20% of the time (Table 4). Moreover, other factors beyond just POS influence training and competition demands and were omitted from the present study for sake of brevity, such as travel and home vs. away vs. neutral site competitions, the quality of an opponent, and player statuses/roles (Fowler et al., 2017; Staunton et al., 2018; Fox et al., 2019, 2021). Future investigations and practitioners aiming to implement similar analysis strategies into their practices should consider these factors (and more) as they begin structuring to their athlete monitoring framework.

In attempt to improve the sample size, an entire season of data were examined in the PCA to increase intraindividual variations in external loads. To further remedy this concern, future research should consider longitudinal studies that venture beyond a single season. This will provide a greater understanding of the external load demands during competition, particularly as it relates to seasonal changes (or perhaps a lack thereof). Moreover, to improve interindividual variance (between player variance), collaborative efforts are encouraged that will allow further investigation into differences across positions, competitive playing levels, and coaching styles. These types of efforts will assist in remedying much of the challenge in sport science, basketball particularly in this case, in which sample sizing might be inherently limited. In the present study, one limitation was the partitioning of players into three POS groups as the sample sizes for each group were then significantly reduced. Consequently, any subsequent predictions based on statistical modeling will only be that much more accurate as the sampling sizes increase. Additionally, the inclusion of an internal load measure (or multiple) overlayed with external load will greatly contribute to this body of knowledge (i.e., load demands during basketball competition and training).

## Practical Applications

Training programs for basketball athletes, especially at the micro- and meso-cycle level of periodization, should consider the varying external load demands during competition on individual athletes because it is likely that the demands differ based on player roles (e.g., POS). Resultantly, the accumulated fatigue from competition demands may differ across POS and warrant individualized recovery and training load modifications throughout the season. Meanwhile, the preparation for the season may also be dependent upon the player roles to ensure each athlete is physiologically equipped to endure the high volumes of decelerations and explosive vertical jumping capabilities, as well as maximal speeds of NCAA DI basketball competitions. These data provide a detailed framework that may help coaches better understand the demands of a collegiate basketball season (e.g., positional differences, physiological demands throughout a season, etc.) and, for those with access to player tracking technology, presents a useful strategy for handling player tracking data.

## Conclusions

The PCA was effective at (1) reducing the number of dimensions in a large, longitudinal, team-sport dataset and (2) identifying external load variables that are sensitive to differences in POS demands during basketball competition. A culmination of summated decelerations, accelerations, jumping and mechanical loads, as well as average and maximal speeds possessed large loadings in the first two components of the PCA (≥ 70). Further analysis revealed that totDEC, totMECH, totJUMP, and maxSPD were the most sensitive to differences in POS external load demands during competition. Therefore, it is recommended to focus on these variables to characterize competition demands, especially for POS groups in DI Power 5 basketball athletes.

## Data Availability Statement

The raw data supporting the conclusions of this article will be made available by the authors, without undue reservation.

## Ethics Statement

The studies involving human participants were reviewed and approved by West Virginia University Institutional Review Board. The patients/participants provided their written informed consent to participate in this study.

## Author Contributions

JS, JM, JR, RB, GC, WH, HS, SG, and JH: conceptualization, writing—original draft, writing—review, and editing. JS and JR: data curation and formal analysis. SG and JH: funding acquisition. JS, JM, JR, RB, GC, SG, and JH: investigation. JS, JR, RB, GC, WH, SG, and JH: methodology. JS, SG, and JH: project administration and resources. JS, RB, and GC: software. JS, RB, SG, and JH: supervision. JS and RB: validation. JS, JM, JR, and JH: visualization. All authors contributed to the article and approved the submitted version.

## Funding

This study was funded internally by the Rockefeller Neuroscience Institute at West Virginia University.

## Conflict of Interest

The authors declare that the research was conducted in the absence of any commercial or financial relationships that could be construed as a potential conflict of interest.

## Publisher's Note

All claims expressed in this article are solely those of the authors and do not necessarily represent those of their affiliated organizations, or those of the publisher, the editors and the reviewers. Any product that may be evaluated in this article, or claim that may be made by its manufacturer, is not guaranteed or endorsed by the publisher.

## References

[B1] Alt P. S. Baumgart C. Ueberschär O. Freiwald J. Hoppe M. W. (2020). Validity of a local positioning system during outdoor and indoor conditions for team sports. Sensors 20:5733. 10.3390/s20205733 33050174 PMC7601858

[B2] Bunker R. P. Thabtah F. (2019). A machine learning framework for sport result prediction. Appl. Comput. Inform. 15, 27–33. 10.1016/j.aci.2017.09.005

[B3] Casamichana D (2019). Looking for complementary intensity variables in different training games in football. J. Strength Condition. Res. 10.1519/JSC.0000000000003025 30844980

[B4] Cohen J (1988). Statistical Power Analysis for the Behavioral Sciences 2nd ed. Hillsdale NJ: Erlbaum.

[B5] Cunanan A. J. DeWeese B. H. Wagle J. P. Carroll K. M. Sausaman R. Hornsby W. G.. (2018). The general adaptation syndrome: a foundation for the concept of periodization. Sports Med. 48, 787–797. 10.1007/s40279-017-0855-3 29307100

[B6] Edwards T. Spiteri T. Piggott B. Bonhotal J. Haff G. G. Joyce C. (2018). Monitoring and managing fatigue in basketball. Sports. 6:19. 10.3390/sports6010019 29910323 PMC5969183

[B7] Federolf P. Reid R. Gilgien M. Haugen P. Smith G. (2014). The application of principal component analysis to quantify technique in sports. Scand. J. Med. Sci. Sports. 24, 491–499. 10.1111/j.1600-0838.2012.01455.x 22436088

[B8] Fowler P. M. Knez W. Crowcroft S. Mendham A. E. Miller J. Sargent C.. (2017). Greater effect of east vs. west travel on jet-lag, sleep and team-sport performance. Med Sci Sports Exerc. 10.1249/MSS.0000000000001374 28719491

[B9] Fox J. L. Green J. Scanlan A. T. (2021). Not all about the effort? A comparison of playing intensities during winning and losing game quarters in basketball. Int. J. Sports Physiol. Perform. 1, 1–4. 10.1123/ijspp.2020-0448 33662929

[B10] Fox J. L. Scanlan A. T. Stanton R. (2017). A review of player monitoring approaches in basketball. J. Strength Condition. Res. 31, 2021–2029. 10.1519/JSC.0000000000001964 28445227

[B11] Fox J. L. Stanton R. Sargent C. O'Grady C. J. Scanlan A. T. (2019). The impact of contextual factors on game demands in starting, semiprofessional, male basketball players. Int. J. Sports Physiol. Perform. 15, 450–456. 10.1123/ijspp.2019-0203 31605525

[B12] Gual G. Fort-Vanmeerhaeghe A. Romero-Rodríguez D. Tesch P. A. (2016). Effects of in-season inertial resistance training with eccentric overload in a sports population at risk for patellar tendinopathy. J. Strength Condition. Res. 30, 1834–1842. 10.1519/JSC.0000000000001286 26670989

[B13] Howatson G. Milak A. (2009). Exercise-induced muscle damage following a bout of sport specific repeated sprints. J. Strength Condition. Res. 23, 2419–2424. 10.1519/JSC.0b013e3181bac52e 19826279

[B14] Jones B. Sall J. (2011). JMP statistical discovery software. Wiley Interdisc. Rev. 3, 188–194. 10.1002/wics.16225855820

[B15] Kaiser H. F (1960). The application of electronic computers to factor analysis. Educ. Psychol. Meas. 20, 141–151. 10.1177/001316446002000116

[B16] Kaiser H. F. Little R. J. (1974). Educational and psychological measurement. SAGE J. 34, 111–117. 10.1177/001316447403400115

[B17] Laffaye G. Wagner P. P. Tombleson T. I. L. (2014). Countermovement jump height: gender and sport-specific differences in the force-time variables. J. Strength Condition. Res. 28, 1096–1105. 10.1519/JSC.0b013e3182a1db03 23838969

[B18] Luo D. Ding C. Huang H. (2011). “Linear discriminant analysis: New formulations and overfit analysis,” in Proceedings of the AAAI Conference on Artificial Intelligence (San Francisco, CA).

[B19] Merrigan J. J. Jones M. T. (2021). Acute inflammatory, cortisol, and soreness responses to supramaximal accentuated eccentric loading. J. Strength Condition. Res. 35, S107–S113. 10.1519/JSC.0000000000003764 33666595

[B20] Merrigan J. J. Stone J. D. Ramadan J. Hagen J. A. Thompson A. G. (2021). Dimensionality reduction differentiates sensitive force-time characteristics from loaded and unloaded conditions throughout competitive military training. Sustainability 13:6105. 10.3390/su13116105

[B21] O'Donoghue P (2008). Principal components analysis in the selection of key performance indicators in sport. Int. J. Perform. Analy. Sport 8, 145–155. 10.1080/24748668.2008.11868456

[B22] Parmar N. James N. Hearne G. Jones B. (2018). Using principal component analysis to develop performance indicators in professional rugby league. Int. J. Perform. Analy. Sport 18, 938–949. 10.1080/24748668.2018.1528525

[B23] Rojas-Valverde D. Gómez-Carmona C. D. Gutiérrez-Vargas R. Pino-Ortega J. (2019). From big data mining to technical sport reports: the case of inertial measurement units. BMJ Open Sport Exerc. Med. 5:e000565. 10.1136/bmjsem-2019-000565 31673403 PMC6797247

[B24] Rojas-Valverde D. Pino-Ortega J. Gómez-Carmona C. D. Rico-González M. (2020). A systematic review of methods and criteria standard proposal for the use of principal component analysis in team's sports science. Int. J. Environ. Res. Public Health. 17:8712. 10.3390/ijerph17238712 33255212 PMC7727687

[B25] Russell J. L. McLean B. D. Impellizzeri F. M. Strack D. S. Coutts A. J. (2020). Measuring physical demands in basketball: an explorative systematic review of practices. Sports Med. 51, 81–112. 10.1007/s40279-020-01375-9 33151481

[B26] Schelling X. Torres-Ronda L. (2016). An integrative approach to strength and neuromuscular power training for basketball. Strength Condition. J. 38, 72–80. 10.1519/SSC.0000000000000219

[B27] Staunton C. Wundersitz D. Gordon B. Custovic E. Stanger J. Kingsley M. (2018). The effect of match schedule on accelerometry-derived exercise dose during training sessions throughout a competitive basketball season. Sports 6:69. 10.3390/sports6030069 30041486 PMC6162803

[B28] Stein M. Janetzko H. Seebacher D. Jäger A. Nagel M. Hölsch J.. (2017). How to make sense of team sport data: from acquisition to data modeling and research aspects. Data 2:2. 10.3390/data2010002

[B29] Stone M. H. Hornsby W. G. Haff G. G. Fry A. C. Suarez D. G. Liu J.. (2021). Periodization and block periodization in sports: emphasis on strength-power training—a provocative and challenging narrative. J. Strength Condition. Res. 35, 2351–2371. 10.1519/JSC.0000000000004050 34132223

[B30] Svilar L. Castellano J. Jukic I. Casamichana D. (2018). Positional differences in elite basketball: selecting appropriate training-load measures. Int. J. Sports Physiol. Perform. 13, 947–952. 10.1123/ijspp.2017-0534 29345556

[B31] Svilar L. Jukić I. (2018). Load monitoring system in top-level basketball team. Kinesiology 50, 25–33. 10.26582/k.50.1.4

[B32] Taylor K. Chapman D. Cronin J. Newton M. J. Gill N. (2012). Fatigue monitoring in high performance sport: a survey of current trends. J. Aust. Strength Cond. 20, 12–23.

[B33] Team R. C (2019). R: A Language and Environment for Statistical Computing. The R Foundation.

[B34] Terner Z. Franks A. (2021). Modeling player and team performance in basketball. Ann. Rev. Statist. Appl. 8, 1–23. 10.1146/annurev-statistics-040720-015536

